# Intestinal Obstruction

**Published:** 1912-09

**Authors:** H. Stokes Munroe

**Affiliations:** Columbus, Ga.


### INTESTINAL OBSTRUCTION.

BY H. STOKES MUNROE, A. B., M. D., COLUMBUS, GA.

The intestinal canal extending from the stomach to the anus is a necessary part of the human anatomy. Parts of it may be removed and if enough is left to properly perform the functions required of it by nature, the individual will live in good health and comfort. Parts of it may be diseased, and the functions greatly impaired, but still life be preserved. But if the lumen of the canal is at any point closed so as to prevent the passage of the intestinal contents, we have a condition no less serious in results than a cessation of the circulation of the blood, and it is only a question of time as to how long the patient will live, unless the obstruction is relieved.

Intestinal obstruction is a serious condition, perhaps more so than any other abdominal lesion with which we have to deal. The mortality is even high with the surgical means at present employed. In my experience, the most difficult and trying cases with which I have had to contend in abdominal surgery have been those of intestinal obstruction. The toxaemia, the early prostration, the malignancies and other factors make these cases poor surgical risks. An early positive diagnosis is not always easily made. There are no pathognomonic symptoms of obstruction on which we can depend in making a diagnosis differentiating it from other acute abdominal troubles. We have been taught that *fecal vomiting* is pathognomonic of intestinal obstruction, but I have never yet seen it in a case. Although it may be a late symptom in many cases, it comes too late to be of value to the diagnostician.

The most prominent symptoms are abdominal pain, paroxysmal in type, excessive peristalsis, nausea and vomiting, the vomited matter at first consisting of gastric contents, later bile and feculant matter; obstipation, although there may be fecal matter passed below the obstruction, and tympanites above the point of obstruction. In some conditons a palpable mass may be found in the abdomen. There is early prostration, no elevation of temperature until late; sometimes visible and palpable waves of peristalsis can be found in the abdomen. If the obstruction is low down the bowels may act frequently, and the actions will consist largely of a watery, floculent mixture, sometimes containing blood. These symptoms, however, are variable, according to the extent of the obstruction.

The prognosis also is variable, as well as the treatment demanded.

It is difficult in most cases before operation to decide upon the exact cause and degree of the obstruction, but our treatment is somewhat dependent upon the classification of the cases.

It is with this in view that I suggest a classification of these cases that will help to clear up some of the complexities of diagnosis and treatment, and I wish to report a typical case illustrative of each type. Various classifications have been given, based on the causes, the anatomical parts involved, and the extent of the trouble. Scientifically, these are good, but for practical purposes the division here suggested is of even more value than any other. This consists of four types:

1. *Paralytic Obstruction.*—In this condition all peristalsis stops. There is an apparent paralysis of the bowels. The bowels will not move. There is nausea and continuous vomiting. The abdomen becomes greatly distended, high enemata and other means are of no avail in moving the gas. The pulse and respiration are rapid, the patient looks anxious, the eyes sunken, and he soon passes into a state of collapse and dies, unless carefully managed. It occurs after septic abdominal operations, with either local or general peritonitis, and is also seen as a late condition in typhoid fever, especially those in which a perforation has occurred. This type is called by most writers "post-operative ileus." Following operations, it is difficult to differentiate from mechanical obstruction. The most important diagnostic points are that in paralytic ileus the symptoms of obstruction develop earlier, usually on the second or third day, and the peristalsis is, as a rule, abolished. A good per cent. of these cases will recover. The best treatment is to wash out the stomach with normal saline solution every three hours until the nausea and vomiting subside. Inject a pint of normal saline in the rectum every three hours. Discard all water and nourishment by mouth. If the patient is not better at the end of thirty-six hours, open the wound and make an opening in one of the distended loops of bowels that first presents itself and insert a large rubber catheter, through which irrigate the bowels at regular intervals with saline solution. I do not approve of the high stimulating enemata used to excess nor of eserine given hypodermically. This tempora ' paralysis of the bowels is nature's method of splinting the abdominal cavity to prevent a spreading peritonitis, and it is not wise to attempt to destroy that splint.

Report of Case. Miss E., age 16, appendiceal abscess opened and drained with large rubber tube on tenth day after onset of attack. Patient progressed nicely first thirty-six hours. Was given two grains of calomel in broken doses. Soon followed by nausea, vomiting, distension of abdomen; bowels failed to move, stimulating enemata had no effect. Used gastric lavage every three or four hours with only temporary relief of nausea. Expression became anxious, eyes sunken, pulse rapid and temperature rose to 102. Her condition appeared desperate, so the wound was opened and an opening made in the caecum; a large rubber catheter was inserted and stitched in position. Through this the colon was irrigated at regular intervals. The gas gradually passed through the opening in the bowel, and the distension and other alarming symptoms soon subsided. The patient made a gradual recovery. The fecal fistula remaining closed spontaneously in about three months.

II. *Partial Obstruction or Stenosis.*—This is caused by constricting bands, contracting cicatrices of ulcerations, tumors of the bowel, foreign bodies, impactions, etc. The obstruction is not complete. The condition is not so desperate. The symptoms are not so distressing. It is in stenosis gradualy developed that the muscles of the bowel above the lesion became greatly hypertrophied by their constant effort to expel the contents of the bowel and through the small opening that we can see and feel the waves of peristalsis on the abdomen.

The pains come in paroxysms, along with the peristaltic movements. There is not the obstinate constipation for the bowels may move daily. Gurgling of the gas and liquid through the small opening can often be heard while palpating the peristaltic movements. There is not the obstinate constipation, nor is the prostration so early and marked. Tympanitis is moderate. All cases are likely to develop into complete obstruction unless the cause is removed. The treatment should be liquid diet, salines in laxatives doses, and high enemata. If the symptoms persist, or if complete obstruction develops, operation is demanded. In suspected malignancy, it should be done as soon as the suspicion arises.

The prognosis for recovery from operation in these cases is good. If the patient is not operated upon early his system should be kept full of normal saline by hypodermoclysis and rectal injections until it can be done.

Report of Case. Mr. I., age 35, one year ago had serious stomach trouble with palpable lump in the stomach. This tumor in the stomach was removed by Dr. Finney, of Baltimore, and pathological examination showed it to be a benign growth. Nine months later patient began to complain of intestinal indigestion, with gas, and colicky pains. The pains gradually became more severe in character and more frequent. He lost weight. I saw him for the first time then. He had severe paroxysms of abdominal pain, visible and palpable peristalsis along right side beginning at caecum and extending to middle of transverse colon. With each of these attacks the gurgling of intestinal contents could be plainly heard. The patient had hiccoughs, slight nausea and his bowels moved naturally several times daily. There was a palpable mass the size of a lemon just below the gall bladder area. This was a plain case of stenosis of the transverse colon due to a growth which had gradually increased in size. He returned to Dr. Finney for advice, but he pronounced the trouble non-operable malignancy and refused to operate. The patient died a few weeks after returning home, from obstruction.

III. *Mechanical Obstruction without Strangulation.*—Most gradually forming, or chronic obstructions, are of this type. Constricting bands, kinks due to adhesion, and foreign bodies may cause obstruction of the bowels without any strangulation. Some very interesting and instructive experiments have recently been performed on dogs by Drs. Hartwell and Hoquett, of Cornell University, and their report was read at the last meeting of the American Medical Association. They proved conclusively that in simple obstruction in dogs, caused by applying a clamp in the upper intestine just tight enough to occlude the bowel but not produce any strangulation or necrosis of the intestine, that the cause of death is depletion of the body—due to the excessive loss of fluid thrown off by the vomiting. If let alone the dogs would invariably die in from three to ten days. But if given normal saline subcutaneously in excess of the fluid vomited and eliminated through the kidneys, the dogs would live in fairly good health for three or four weeks, which was as long as the experiments continued. The stomachs were also washed out regularly until the vomiting ceased. It took about one-tenth of the body weight of the animal in normal saline given daily to supply the loss. At the end of the three weeks the clamps were removed in several cases and the dogs made uninterrupted recoveries. The same results, however, they do not claim will apply to cases in which there is present strangulation of the bowels, for in such cases there is some toxin produced in the damaged bowel, bacterial or chemic in origin, which plays an important role in the death of the animal, regardless of the saline treatment. We draw the conclusion from the experimental reports, that in simple obstruction without strangulation, the patient's condition can be improved for operation by the saline treatment and gastric lavage. The saline, however, should be given in large amounts; certainly in excess of the quantity of fluids thrown off from the body by both stomach and kidneys. Operation is the only curative treatment for this and should invariably be done as soon as the patient is in condition for it. It is, however, not always an easy matter to say whether or not the obstruction is simple or complicated by some interference with the circulation of the bowel.

Report of Case. Mrs. P., aged 37, nine years previous had ovaries and tubes removed for inflammatory troubles. This was followed by large pelvic abscess which drained through the vagina; since that time health had not been good—suffered from chronic constipation and intestinal disorders; several months ago was taken suddenly with nausea. Began to vomit and vomited incessantly everything swallowed. A few days later pains began in the abdomen accompanied by violent peristaltic movements. The location of the pain was variable, but usually referred to the right iliac region. Pulse ranged around 100. Temperature was at first normal, but in a few days ran to 100. No abdominal rigidity; considerable distension for few days; diagnosis of preliminary stenosis with acute obstruction developing. The patient was given large amount of normal saline per rectum, containing liquid peptonoid. The stomach pump was used occasionally and the stomach kept empty. On the ninth or tenth day, after a gallon of saline solution was injected into the rectum, a large quantity of featus was expelled. Following this, the bowels were with difficulty moved daily. Her condition improved and a few days later an exploratory incision was made through the right rectus muscle. The appendix had slight adhesions, but was otherwise normal. The hepatic flexure of the colon and part of the ascending and transverse colon were firmly bound down with bands of adhesions and surrounded by firm attachments to the omentum. The adhesions were all loosened up and the bowel freed after a long and tedious dissection. Abdomen closed without drainage. The patient convalesced slowly and in several weeks was up and about. There was still some pain and tenderness in the abdomen. The bowels moved regularly and with comparative ease. She did nicely for about two months and again began to vomit and have severe pains. She was so much weakened by her previous experience that we did not wait on conditions but at once advised operation for resection of the diseased part. The adhesions around the colon were so dense and firm that it was another long, tedious dissection to free it, and two small perforations were made in the bowels during the manipulation. The patient's condition was not good at the end of the dissection, so we did not remove the section, but made a lateral anastomosis around it between the jejunum and transverse colon. Her conditon was fairly good for the first forty-eight hours, when her pulse became more rapid, pains in the abdomen increased, and the abdomen became very much distended. She died in about twenty-four hours. No post-mortem was done, so I do not know the exact cause of her death. I put free drainage in the wound, but there was either a septic peritonitis or insufficient relief of the obstruction. I never could determine the cause of those massive adhesions, there was nothing in the history pointing to previous inflammation so high up in the abdomen.

IV. *Mechanical Obstruction with Strangulation*—This is the most serious type of obstruction and without early surgical intervention the patient will probably die. Whenever the circulation of the bowel is impaired there is some toxin produced and readily absorbed that acts powerfully in depressing the patient. This is the type of obstruction produced by volvulus, strangulated hernia, intussusception, adherent bands around loops of intestine, emboli in mesenteric vessels, causing gangrene of sections of the bowels and other such mechanical causes. Prompt diagnosis and surgical intervention is the treatment and the only treatment of avail. If late in operating it is always wise to wash out the interior of the strangulated loop, and be sure no weak or gangrenous parts are left.

Report of Case. Mrs. P., age 72, had small femoral hernia for ten years, which had given no serious trouble. Early in the morning, soon after arising, she was stricken with sudden acute pain at the site of the hernia. This was followed by severe pains in the lower abdomen, nausea and vomiting. The lump in the groin was tender and could not be reduced by taxis. Diagnosis—strangulated femoral hernia. We operated on her that afternoon with local anaesthesia. A loop of small intestine four inches long, almost black in color and tightly constricted at the femoral ring was found. Also a small mass of omentum firmly adherent to the sac. After relieving the constriction and applying hot compresses to the bowel for ten or fifteen minutes, the color gradually returned to the bowel and the operation was completed. She made a rapid recovery with no complications.

For mechanical obstruction with strangulation the important thing in treatment is early operation. The mortality after late operations is high. Every hour of temporizing the patient is growing worse. In the first three varieties it may be safe and wise to wait if the exact condition is recognized and the patient properly managed.

